# Highly Efficient Oxygen Evolution Reaction Enabled by Phosphorus Doping of the Fe Electronic Structure in Iron–Nickel Selenide Nanosheets

**DOI:** 10.1002/advs.202101775

**Published:** 2021-07-24

**Authors:** Yuan Huang, Li‐Wen Jiang, Bu‐Yan Shi, Kevin M. Ryan, Jian‐Jun Wang

**Affiliations:** ^1^ Institute of Crystal Materials, State Key Laboratory of Crystal Materials, Shenzhen Research Institute of Shandong University Shandong University Jinan Shandong 250100 China; ^2^ Department of Chemical Sciences and Bernal Institute University of Limerick Limerick Ireland

**Keywords:** electronic structure, Ni_0.75_Fe_0.25_Se_2_, oxygen evolution reaction, P doping

## Abstract

The electronic structure of active sites is critically important for electrochemical reactions. Here, the authors report a facile approach to independently regulate the electronic structure of Fe in Ni_0.75_Fe_0.25_Se_2_ by P doping. The resulting electrode exhibits superior catalytic performance for the oxygen evolution reaction (OER) showing a low overpotential (238 mV at 100 mA cm^−2^, 185 mV at 10 mA cm^−2^) and an impressive durability in an alkaline medium. Additionally, the mass activity of 328.19 A g^−1^ and turnover frequency (TOF) of 0.18 s^−1^ at an overpotential of 500 mV are obtained for P─Ni_0.75_Fe_0.25_Se_2_ which is much higher than that of Ni_0.75_Fe_0.25_Se_2_ and RuO_2_. This work presents a new strategy for the rational design of efficient electrocatalysts for OER.

## Graphical abstract

The precise electronic structure regulation of Fe in Ni_0.75_Fe_0.25_Se_2_ by P doping leads to the P─Ni_0.75_Fe_0.25_Se_2_ electrode with remarkable electrocatalytic activity and stability for oxygen evolution reaction (OER).

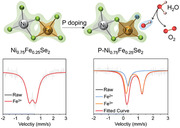

## 1 Introduction

Electrochemical water splitting using intermittent renewable energy is a highly attractive approach for producing hydrogen without CO_2_ emission.^[^ ^1^, ^2^, ^3^ ^]^ The anodic oxygen evolution reaction (OER) is kinetically sluggish due to the four proton‐coupled electron transfer kinetics and the oxygen─oxygen bond formation.^[^ ^4^, ^5^, ^6^, ^7^ ^]^ Currently, noble metal‐based catalysts such as iridium and ruthenium oxides (IrO_2_ or RuO_2_) are recognized as the most active OER catalysts although as precious metals, their cost and low earth abundance makes the technology competitively unviable against fossil fuels.^[^ ^8^ ^]^ Recently, transition metal‐based (ranging from metal oxides, phosphides, chalcogenides, to emerging single‐atom) catalysts have been considered as promising candidates to replace noble metal‐based catalysts for OER due to their low cost, excellent activity, and high stability.^[^ ^9^, ^10^, ^11^, ^12^ ^]^ In particular, considerable efforts have been devoted to developing nickel‐iron‐based OER electrocatalysts.^[^ ^7^, ^13^, ^14^ ^]^ Among them, NiFe‐selenides have been widely investigated as ideal OER candidates due to the high electronic conductivity, diversity of stable crystal phases, and adjustable electronic structure.^[^ ^15^, ^16^ ^]^ Additionally, engineering the porosity, selenium vacancy, and the polarized electronic spin of Fe/Ni further enables the optimization of absorption/desorption of reaction intermediates and gas release.^[^ ^17^, ^18^, ^19^, ^20^ ^]^

Crucially, although the nickel–iron (Ni–Fe)‐based bimetal electrocatalysts exhibit remarkable OER performance, there is still no scientific consensus on whether nickel or iron is the active center.^[^ ^21^ ^]^ According to the Sabatier principle, the studies on metal hydr(oxy)oxides suggest that Ni might be the active site due to the optimal interaction strength with OH_ad_.^[^ ^22^ ^]^ The presence of Fe is thought to affect the charge contribution leading to high valence Ni cations thereby enhancing their OER performance.^[^ ^23^ ^]^ Indeed, the absorption of Fe impurities was reported to exert a partial‐charge transfer activation effect on Ni improving the conductivity of Ni based electrocatalysts.^[^ ^24^ ^]^ The study by Hu et al. revealed that Fe sites dominate the catalysis and the activity of Fe sites are 20–200 times higher than those of Ni sites in NiFe layered double hydroxides (LDHs).^[^ ^25^ ^]^ In related work, Chen et al. proposed that Fe^4+^ species are not directly responsible for the OER activity but the theoretical results suggested that high spin Fe^4+^ leads to efficient formation of an active O radical intermediate. They further suggest that Ni^4+^ catalyzes the subsequent O─O coupling, and it is the synergy between Fe and Ni that is responsible for the optimal performance for OER.^[^ ^26^, ^27^ ^]^ The results to‐date show that the electronic structures of Ni and Fe are integral to the mechanism which drives enhanced OER activity and understanding and tuning these electronic structures is the key to unlocking the pathways involved. More recently, elemental doping (Co, Fe, etc.) has also emerged as an efficient strategy to regulate the electronic structure of target materials.^[^ ^18^, ^20^, ^28^, ^29^, ^30^, ^31^, ^32^, ^33^ ^]^ In this study, we have developed a facile approach to independently regulate the electronic structure of Fe in Ni_0.75_Fe_0.25_Se_2_ nanosheets by P doping.

## 2 Results and Discussion

**Figure 1:**
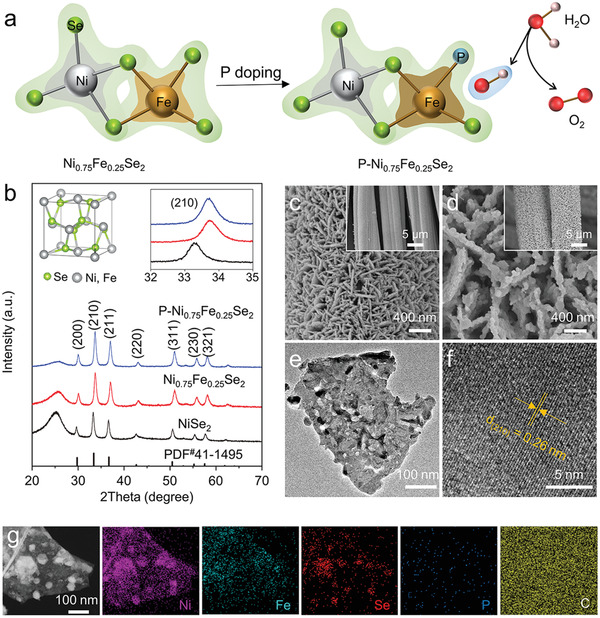
a) Schematic diagram of the electronic structure changes for Ni_0.75_Fe_0.25_Se_2_ before and after P doping, the shaded part signifies the electronic cloud. b) XRD patterns and c,d) SEM of Ni_0.75_Fe_0.25_Se_2_ and P─Ni_0.75_Fe_0.25_Se_2_. The insets in (b) show the unit cell of cubic metal diselenides and the magnified XRD patterns. The insets in Figure 1c,d shows the overall morphology of Ni_0.75_Fe_0.25_Se_2_ and P─Ni_0.75_Fe_0.25_Se_2_ on carbon cloth, respectively. e) TEM, f) high‐resolution TEM image, and g) STEM image and corresponding elemental mapping images of P─Ni_0.75_Fe_0.25_Se_2_.

A schematic of Ni_0.75_Fe_0.25_Se_2_ and its P doped analogue P─Ni_0.75_Fe_0.25_Se_2_ is outlined in **Figure** **1a** with the synthetic route described in Scheme S1, Supporting Information. Briefly, hexagonal NiFe layered double hydroxide (LDH) (Ni_0.75_Fe_0.25_(CO_3_)_0.125_(OH)_2_·0.38H_2_O) with a lateral size of ≈15 nm (Figures S2–S5, Supporting Information) is transformed into cubic Ni_0.75_Fe_0.25_Se_2_ by selenization. The X‐ray power diffraction (XRD) peaks at 30.1°, 33.7°, 37.1°, 43.0°, 50.9°, 55.6°, 58.0°, and 62.4° (Figure 1b) can be ascribed to the (200), (210), (211), (220), (311), (230), (321) crystallographic planes of cubic Ni_0.75_Fe_0.25_Se_2_ (JCPDS No. 41–1495), respectively_._^[^ ^34^ ^]^ The broad peak at around 25° is ascribed to the carbon cloth substrate (Figure S3, Supporting Information).^[^ ^35^, ^36^ ^]^ Compared with NiSe_2_, the peaks were shifted to higher angles due to the incorporation of Fe, further confirming the formation of Ni_0.75_Fe_0.25_Se_2_ (Figure 1b‐inset).^12^ It is worth noting that the crystal structure remains unchanged after P doping (Figure 1b). Notably, the interlinked nanosheet structure was preserved after doping with the thickness of nanosheets increasing slightly to ≈50 nm (Figure 1c,d and Figures S6 and S7, Supporting Information). Additionally, a small number of nanoparticles with a diameter of ≈30–50 nm is evident on the surface of the nanosheet (Figure 1d and Figure S7, Supporting Information). The observation by transmission electron microscope (TEM, Figure 1e) is consistent with the scanning electron microscopy (SEM) observation. The d‐spacing of 2.6 Å (Figure 1f) can be well indexed to the (210) plane of cubic Ni_0.75_Fe_0.25_Se_2_. The elemental mapping images (Figure 1g and Figure S8, Supporting Information) corroborated that the existence and homogeneous distribution of Ni, Fe, Se, and P elements within the sample. The composition was further determined by the inductively coupled plasma analysis spectrometry (ICP, Table S1, Supporting Information) to be Fe/Ni/Se/P∼1/3/8/0.03.

**Figure 2:**
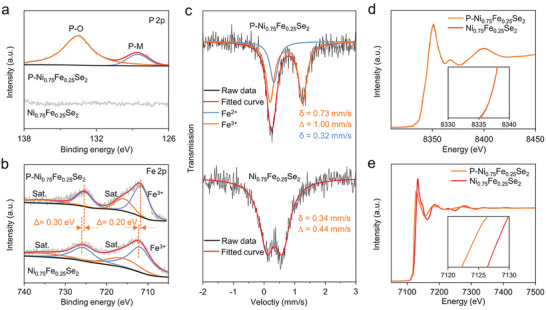
Electronic structure characterizations of Ni_0.75_Fe_0.25_Se_2_ and P─Ni_0.75_Fe_0.25_Se_2_: a,b) high‐resolution XPS spectra: a) P 2p, b) Fe 2p. c) Mössbauer spectra. d,e) XANES spectra: d) Ni K‐edge, e) Fe K‐edge.

Compared with the X‐ray photoelectron spectroscopy (XPS) of Ni_0.75_Fe_0.25_Se_2_, the presence of peaks located at 128.6 and 133.4 eV (**Figure** **2a**) were assigned to P─*M* (Fe or Ni) and P─O bands due to surface oxidation, respectively, further confirming that P was successfully doped into Ni_0.75_Fe_0.25_Se_2_.^[^ ^37^, ^38^ ^]^ The P content determined by XPS (Table S2, Supporting Information) is much higher than the value obtained by the ICP examination, suggesting that the P element is mainly distributed close to the surface. In the region of Fe 2p (Figure 2b), the peaks can be fitted with two prominent peaks at 711.7 and 725.4 eV with two satellite peaks, indicative of Fe^3+^.^[^ ^17^, ^37^, ^39^ ^]^ After P doping, the two main peaks of Fe^3+^ were shifted slightly to a lower binding energy, while the spectrum of Ni 2p showed negligible change (Figure S9, Supporting Information), suggesting that the electronic structure of Fe was modulated by introducing P. To confirm the modulation effect, Mössbauer analysis was applied and the results in Figure 2c reveal that a doublet with an isomer shift (*δ*) of 0.34 mm s^−1^ and quadrupole splitting (*Δ*) of 0.44 mm s^−1^ for Ni_0.75_Fe_0.25_Se_2_ were observed, confirming the high‐spin, Jahn–Teller‐distorted Fe^3+^ species, similar to those reported previously.^[^ ^26^ ^]^ As for P─Ni_0.75_Fe_0.25_Se_2_, the doublet peaks can be fitted with the signals of Fe^2+^ (*δ* = 0.32 mm s^−1^) and high spin Fe^3+^ (*δ* = 0.73 mm s^−1^ and *Δ* = 1.00 mm s^−1^).^[^ ^40^, ^41^ ^]^ The Mössbauer data with the XPS results proves that P doping in Ni_0.75_Fe_0.25_Se_2_ can enrich the electron cloud around Fe^3+^. The electronic structure of Fe and Ni was further investigated by X‐ray absorption spectra (XAS). The Ni‐K edge X‐ray absorption near‐edge spectra (XANES) of the samples before and after P doping completely overlaps, indicating that the electronic structure of Ni remained unchanged after P doping (Figure 2d). Furthermore, the corresponding Fourier‐transformed k^3^‐weighted *χ*(k) function (Figure S10, Supporting Information) also signifies that the bonding environment of Ni atom is basically unchanged with obvious peaks at 2.44 and 1.62 Å corresponding to Ni─Se/Fe/Ni bonds and Ni─O bond, respectively.^[^ ^20^, ^42^ ^]^ For the Fe K‐edges XANES (Figure 2e), the curve of P─Ni_0.75_Fe_0.25_Se_2_ shifts to lower energy than Ni_0.75_Fe_0.25_Se_2_, indicating P doping can significantly reduce the valency of Fe^3+^. Another observation is that the peak intensity of Fe‐Se/Fe/Ni for P─Ni_0.75_Fe_0.25_Se_2_ decreases significantly compared with Ni_0.75_Fe_0.25_Se_2_ (Figure S11, Supporting Information). These results imply that P bonds more readily with Fe than Ni and that P‐doping causes severe surface structural disorder.^[^ ^20^, ^42^, ^43^ ^]^

**Figure 3:**
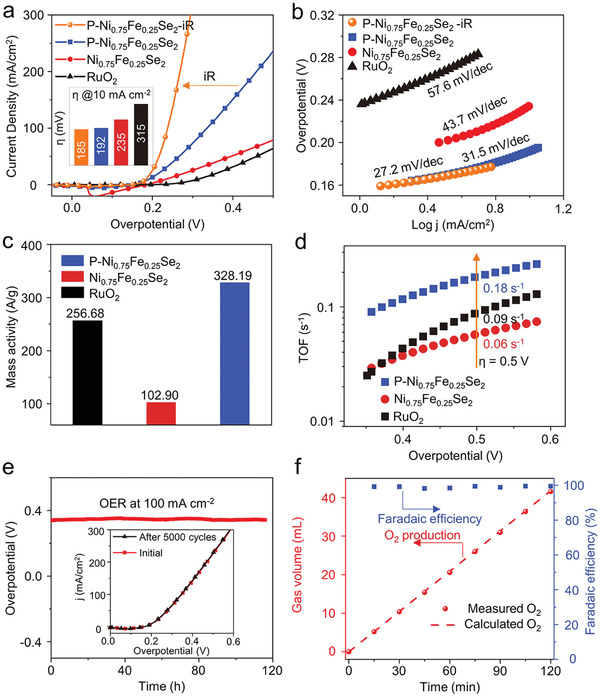
a) OER polarization curves without iR‐correction and the LSV curve of P─Ni_0.75_Fe_0.25_Se_2_ with iR‐correction (orange). The inset shows the overpotentials. b) Tafel pots. c) Mass activity (*η* = 0.50 V) and d) TOF. e) Stability measurements for P─Ni_0.75_Fe_0.25_Se_2_. f) Faradic efficiency.

The linear sweep voltammetry (LSV) polarization curves are displayed in **Figure** **3a**. P─Ni_0.75_Fe_0.25_Se_2_ exhibits significantly enhanced OER activity compared with Ni_0.75_Fe_0.25_Se_2_ and RuO_2_. It needs only a low overpotential (*η*) of 192 mV to drive a current density of 10 mA cm^−2^, which is 20% lower than Ni_0.75_Fe_0.25_Se_2_ and 60% lower than RuO_2_ (the inset in Figure 3a). For Tafel analyses of the catalysts to evaluate the electrocatalytic kinetics (Figure 3b), P─Ni_0.75_Fe_0.25_Se_2_ exhibits a substantially smaller Tafel slope of 31.5 mV dec^−1^ than Ni_0.75_Fe_0.25_Se_2_ (43.7 mV dec^−1^) and RuO_2_ (57.6 mV dec^−1^), indicating faster kinetics. In order to rule out the effect of the contact resistance and compare the performance of P─Ni_0.75_Fe_0.25_Se_2_ with the reported electrocatalysts, iR‐correction was applied for P─Ni_0.75_Fe_0.25_Se_2_ (Figure 3a,b). P─Ni_0.75_Fe_0.25_Se_2_‐iR delivers a current density of 10 mA cm^−2^ at an ultra‐low overpotential of 185 mV with a small Tafel slope of 27.2 mV dec^−1^, suggesting an impressively higher OER catalytic activity than related electrocatalysts (Table S3, Supporting Information). The mass activity (at *η* = 0.50 V) of P─Ni_0.75_Fe_0.25_Se_2_ is 328.19 A g^−1^, which is 3.19 times higher than that of Ni_0.75_Fe_0.25_Se_2_ (102.90 A g^−1^) and 1.28 times RuO_2_ (256.68 A g^−1^) suggesting P‐doping greatly improves the OER activity of Ni_0.75_Fe_0.25_Se_2_ (Figure 3c and Table S4, Supporting Information). Similarly, the TOF in Figure 3d shows that the value of P─Ni_0.75_Fe_0.25_Se_2_ is the largest within the studied potential range, indicating its outstanding intrinsic electrocatalytic activity. These experimental results distinctly demonstrate that regulating the electronic structure of Fe in Ni_0.75_Fe_0.25_Se_2_ by P doping is a viable route to improve its OER catalytic activity. The work further confirms the active role of Fe in Ni_0.75_Fe_0.25_Se_2_ for OER.

The durability of P─Ni_0.75_Fe_0.25_Se_2_ was evaluated by chronopotentiometric measurement at 100 mA cm^−2^ (Figure 3e). The current remained steady for 120 h without any appreciable increase in potential and the LSV curve of P─Ni_0.75_Fe_0.25_Se_2_ remains unchanged after 5000 scans (The inset in Figure 3e). Additionally, the XRD patterns shows no change compared with the initial P─Ni_0.75_Fe_0.25_Se_2_ (Figure S12, Supporting Information), suggesting that no new crystal was formed under the OER stability test. The XPS results indicate that the binding energy of Fe 2p and Ni 2p was shifted slightly to higher energy due to the formation of metal oxide/hydroxide species on the surface of P─Ni_0.75_Fe_0.25_Se_2_ during the OER process (Figure S13, Supporting Information).^[^ ^12^, ^17^, ^20^, ^38^ ^]^ Similarly, the intensity of the P─*M* peak decreased significantly to form P─O species. These in‐situ generated species contribute to the enhanced stability.^[^ ^44^ ^]^ Importantly, the P─Ni_0.75_Fe_0.25_Se_2_ electrode delivered a Faradaic efficiency of ≈100% for OER (Figure 3f), indicating that the observed current exclusively originated from OER rather than other processes.

**Figure 4:**
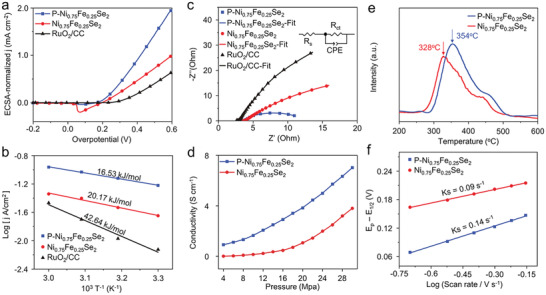
a) ECSA‐normalized LSV curves. b) Arrhenius plots (*η* = 300 mV). c) Nyquist plots, the inset is the equivalent circuit. d) Pressure dependent conductivity. e) O_2_‐TPD curve and f) *K*_s_ of P─Ni_0.75_Fe_0.25_Se_2_ and Ni_0.75_Fe_0.25_Se_2_.

The electrochemical double‐layer capacitance (*C*_dl_) of P─Ni_0.75_Fe_0.25_Se_2_ (6.25 mF cm^−2^) is higher than RuO_2_ (5.92 mF cm^−2^) and Ni_0.75_Fe_0.25_Se_2_ (4.13 mF cm^−2^), indicating P doping indeed increases the number of active sites, which is beneficial for the OER process (Figure S14, Supporting Information). When the LSV curves are normalized by the electrochemical surface area (ECSA) to exclude the contribution of larger ECSA for OER performance, the results shown in **Figure** **4a** indicate that P─N_0.75_Fe_0.25_Se_2_ shows much better OER activity than RuO_2_ and Ni_0.75_Fe_0.25_Se_2_ samples, reflecting that the enhanced OER activity is not only attributed to the increased ECSA but also the improved intrinsic activity of the catalyst due to the optimized electronic structure.^[^ ^45^ ^]^ In addition, the P doping showed minimum influence on the morphology of Ni_0.75_Fe_0.25_Se_2_, confirmed by the comparable surface area before and after P doping (Figure S15a,b, Supporting Information).^[^ ^46^, ^47^ ^]^ The LSV curves normalized by the BET surface area further confirmed the improved intrinsic catalytic activity by P doping (Figure S15c, Supporting Information). The activation energy (*E*_a_) of different catalysts was calculated by measuring the currents at different temperatures (Figure S16, Supporting Information).^[^ ^48^, ^49^ ^]^ Figure 4b shows that the *E*_a_ derived from the slopes of the Arrhenius plot is 16.53 kJ mol^−1^ for P─Ni_0.75_Fe_0.25_Se_2_, much lower than Ni_0.75_Fe_0.25_Se_2_ (20.17 kJ mol^−1^) and RuO_2_ (42.64 kJ mol^−1^), indicating the high catalytic activity of P─Ni_0.75_Fe_0.25_Se_2_. The Nyquist plots (Figure 4c and Table S5, Supporting Information) reveal that the solution resistance (*R*_s_) exhibit negligible change for different catalysts, while the charge–transfer resistance (*R*_ct_) for P─Ni_0.75_Fe_0.25_Se_2_ (8.55 Ω) is reduced by 20 times and 5 times compared to RuO_2_ (165.00 Ω) and Ni_0.75_Fe_0.25_Se_2_ (42.60 Ω), respectively, indicating a faster charge–transfer kinetics between P─NP─Ni_0.75_Fe_0.25_Se_2_ and the electrolyte during the OER process, consolidating the results of the Tafel slopes. The study of the conductivity in Figure 4d shows that P doping to P─Ni_0.75_Fe_0.25_Se_2_ significantly enhances the conductivity and consequently facilitates charge transfer between the current collector and the catalyst during OER, consistent with the EIS results. In addition, the temperature‐programmed desorption of O_2_ (O_2_‐TPD) curve was used to characterize the adsorption strength of O_2_ on the surface of electrocatalysts. N_0.75_Fe_0.25_Se_2_ requires a lower temperature (328 °C) for desorption than that of P─N_0.75_Fe_0.25_Se_2_ (354 °C), suggesting a faster O_2_ desorption process on the surface of Ni_0.75_Fe_0.25_Se_2_ than P─Ni_0.75_Fe_0.25_Se_2_ (Figure 4e). On the other hand, the capability of adsorption and desorption for the oxygen‐containing intermediates (*OH, *O, *OOH) is comparably or even more critical for the performance of OER electrocatalysts.^[^ ^21^, ^22^, ^50^, ^51^ ^]^ The adsorption strength of OH^−^ ions on Ni_0.75_Fe_0.25_Se_2_ and P─Ni_0.75_Fe_0.25_Se_2_ during OER was further verified based on the Laviron analysis.^[^ ^52^ ^]^ As shown in Figure 4f and Figures S17 and S18, Supporting Information, P─Ni_0.75_Fe_0.25_Se_2_ exhibits a larger *K*_s_ (0.14 s^−1^) than Ni_0.75_Fe_0.25_Se_2_ (0.09 s^−1^), suggesting that the enhanced adsorption capability of P─Ni_0.75_Fe_0.25_Se_2_ sites for OH^*^ intermediate facilitates the OER process,^[^ ^52^, ^53^, ^54^ ^]^ in good agreement with the results of O_2_‐TPD.

## 3 Conclusion

We have proposed a unique way to independently regulate the electronic structure of Fe in Ni_0.75_Fe_0.25_Se_2_ by P doping. The optimized electronic structure of the resulting catalyst has been studied and confirmed using XPS, Mössbauer spectra, and XANES spectra. The corresponding electrode exhibits outstanding OER activity and durability to achieve a benchmark current density of 10 mA cm^−2^ at an ultralow overpotential of 185 mV. The mechanistic investigation reveals that P doping endows the Ni_0.75_Fe_0.25_Se_2_ electrocatalyst with enhanced conductivity, optimized adsorption of oxygen‐containing intermediates, and a reduced kinetic barrier. This work provides an in‐depth insight into understanding the effect of P doping in Ni_0.75_Fe_0.25_Se_2_. The use of doping to regulate the electronic structure of a single metal site in multinary transition metal electrocatalysts opens new pathways for enhancement of OER in a wide range of systems.

## Conflict of Interest

The authors declare no conflict of interest.

## Supporting information

Supporting Information

## Data Availability

Research data are not shared.
