# Supplementary material for: Highly Efficient Oxygen Evolution Reaction Enabled by Phosphorus Doping of the Fe Electronic Structure in Iron–Nickel Selenide Nanosheets
Source: Adv Sci (Weinh). 2021 Jul 24;8(18):2101775. doi: 10.1002/advs.202101775 (PMC8456200; doi:10.1002/advs.202101775)
Supplement: Supplementary file 1 — Supporting Information [file ADVS-8-2101775-s001.pdf]

© 2021 Wiley-VCH GmbH

ADVANCED SCIENCE
Open Access

Supporting Information

for *Adv. Sci.*, DOI: 10.1002/advs.202101775

Highly Efficient Oxygen Evolution Reaction enabled by Phosphorus Doping of the Fe Electronic Structure in Iron–Nickel Selenide Nanosheets

*Yuan Huang, Li-Wen Jiang, Bu-Yan Shi, Kevin M. Ryan, Jian-Jun Wang**

Supporting Information

Highly Efficient Oxygen Evolution Reaction enabled by Phosphorus Doping of the Fe Electronic Structure in Iron–Nickel Selenide Nanosheets

Yuan Huang, Li-Wen Jiang, Bu-Yan Shi, Kevin M. Ryan, Jian-Jun Wang*

Experimental Section/Methods

Chemicals and Materials: Carbon cloth (CC) was purchased from Tai Wan CeTech, Taiwan, China. $Ni(NO_3)_2 \cdot 6H_2O$, $FeSO_4 \cdot 7H_2O$, NH_4F, $NaBH_4$, KOH, NaH_2PO_2, urea, ethanol and acetone were obtained from National Reagent Company. Se Powder, commercial RuO_2 and Nafion (5wt%) were provided by Sigma-Aldrich. Milli-Q water (18.2 MΩ cm at 25 ^oC) was used for all the experiments. All reagents are of analytical grade and were used without further purification.

Preparation of NiFe LDH: NiFe LDH precursor was synthesized by using a simple hydrothermal reaction. Typically, $Ni(NO_3)_2 \cdot 6H_2O$ (1 mM), $FeSO_4 \cdot 7H_2O$ (0.4 mM), NH_4F (5 mM) and urea (8 mM) were dissolved in 40 mL deionized water under vigorous stirring to obtain a uniform solution. A piece of cleaned carbon cloth (1 × 3 cm^2) and the aforementioned solution were transferred into a 50 mL Teflon-lined stainless-steel autoclave and maintained at 120 ^oC for 16 h. The NiFe LDH nanosheet array directly grown on CC was taken out and washed with water and ethanol thoroughly for several times, followed by drying at 50 ^oC overnight.

Preparation of $Ni_{0.75}Fe_{0.25}Se_2$: The NaHSe solution was prepared by adding Se powder (65 mg) into deionized water (30 mL) containing $NaBH_4$ (65 mg) under N_2 flow under stirring. In a typical process, the solution was transferred into 50 mL autoclave with a piece of NiFe LDH on carbon cloth and maintained at 180 ^{o}C for 20 h in an oven. The obtained $Ni_{0.75}Fe_{0.25}Se_2$ was taken out and washed with water and ethanol thoroughly for several times, followed by drying at 50 ^{o}C overnight.

Preparation of P-$Ni_{0.75}Fe_{0.25}Se_2$: Generally, 3 mM NaH_2PO_2 was dissolved into 30 mL of deionized water under magnetic stirring. A piece of $Ni_{0.75}Fe_{0.25}Se$ sample was dipped into the aforementioned precursor solution for 10 min and was subsequently taken out. After drying under 50 ^{o}C, the obtained $Ni_{0.75}Fe_{0.25}Se$ was heated at 500 ^{o}C for 1 h with a heating rate of 5 ^{o}C min^{-1} in Ar atmosphere, and then cooled down to room temperature naturally. The final product was defined as P-$Ni_{0.75}Fe_{0.25}Se_{2.}$ The content of phosphorus can be accurately controlled by tuning both the concentration of NaH_2PO_2 solution and the soaking time. The P content was determined by the Inductively Coupled Plasma (ICP) analysis. Typically, a sample of P-$Ni_{0.75}Fe_{0.25}Se$ (1 × 3 cm^2) was dissolved by 10 mL nitric acid solution (5% volume HNO_3 in water, ultrapure) and then the substrate of carbon cloth was taken out. This solution is then filtered through membrane filters to remove the solid material prior to use for ICP analysis.

Characterizations: The crystal structure was characterized by X-ray power diffraction on a Bruker D8 Advance Powder X-ray diffractometer at 40 kV and 40 mA for monochromatized Cu K_{α} (λ = 0.15406 nm). The morphology, composition and structure were studied using Field emission scanning electron microscopy (FESEM, HITACHI S-4800) and transmission electron microscopic with a JEOL JEM 2100 microscope operating at 200 kV. The X-ray photoelectron spectroscopy (XPS) measurements were conducted on the Thermo Scientific ESCALab 250 using 200 W monochromated Al K_{α} radiation. Inductively Coupled Plasma analysis was carried out through the instrument of Thermo iCAP 7200 ICP-OES. The Mössbauer measurements were performed at room temperature using SEE Co W304

Mössbauer spectrometer. A $^{57}Co(Rh)$ source with activity of 25 mCi was used and the velocity calibration was done with a room temperature α-Fe absorber. The spectra were fitted by using the MossWinn 4.0 software. The X-ray absorption fine structure spectra (Ni K-edge and Fe K-edge) were collected at BL14W beamline in Shanghai Synchrotron Radiation Facility (SSRF). The storage rings of SSRF was operated at 3.5 GeV with a stable current of 200 mA. Using Si (111) double-crystal monochromator, the data collection was carried out in fluorescence mode using Lytle detector. All spectra were collected in ambient conditions. Brunauer-Emmet-Teller (BET) specific surface area of samples were determined by Kubo X1000 instrument with nitrogen adsorption at 77 K. The pressure-dependent conductivity was measured by the Semiconductor resistivity of the powder tester (ST-2722, China). The oxygen temperature programmed desorption (O_2-TPD) was conducted on an AutoChem II 2920 chemisorption analyzer (Micromeritics, USA). For a typical O_2-TPD measurement, about 100 mg of each sample was heated to 150 °C under pure He at a heating rate of 10 °C min^{-1}, and kept at this temperature for 1 h. After cooling the sample to room temperature, adsorption of O_2 was carried out in flowing O_2 (ultrahigh purity) for 1 hour. Then switch the gas to pure Ar for 30 min until the baseline is stable. Finally, the O_2-TPD measurement was carried out in flowing pure He with a heating rate of 10 °C min^{-1} until 700 °C.

Electrochemical Measurements: All the electrochemical tests were performed in a standard three-electrode system on an electrochemical workstation (CHI660E, Shanghai Chen Hua Instruments Co. China) with a platinum plate and double salt bridge Ag/AgCl electrode as counter and reference electrodes, respectively. The LSV curves for all the electrocatalysts were recorded after 20 cycles of cyclic voltammetry (CV) until a stable CV curve was obtained. The linear sweep voltammetry curves were recorded in KOH solution (1 M) at a scan rate of 5 mV s^{-1} without iR-correction. The electrochemical impedance spectroscopy (EIS) measurements of all catalysts were conducted in 1 M KOH at 1.43 V (V vs RHE), over a frequency range of 0.01-10^5 Hz and AC amplitude of 5 mV. The 5000 cycles stability test was carried out at a scan rate of 50

mV s^{-1}. The long-term durability test was performed using chronopotentionmetry method at a constant current density of 100 mA cm^{-2}. The C_{dl} values for the as-prepared working electrodes were determined from the cyclic voltammogram (CV) in the double layer region (without faradaic processes) at different scan rates. The Faradic efficiency test of P-$Ni_{0.75}Fe_{0.25}Se_2$ was carried out at a overpotential of 335 mV.

The values of mass activity (A g^{-1}) were calculated from the catalyst loading m (mg cm^{-2}) and the measured current density j (mA cm^{-2}) at $\eta = 0.50$ V:

$$mass\ activity\ = {}^{j}/_{m}$$

The values of TOF were calculated by assuming that every metal atom is involved in the catalysis (lower TOF limits were calculated):

$$TOF\ = \frac{j * S}{4 * F * n}$$

Here, j (mA cm^{-2}) is the measured current density, S (1.00 cm^2) is the surface area of as-prepared working electrodes, the number 4 means 4 electrons per mole of O_2, F is Faraday's constant (96485.3 C mol^{-1}), and n is the moles of the metal atom on the electrode calculated from m and the molecular weight of the coated catalysts.

In order to exclude the influence of ECSA on the performance comparison, the OER curves were normalized by ECSAs. The ECSA-normalized current density for as-prepared catalysts was calculated by:

$$\text{ECSA-normalized current density} = \text{current density} \times C_s/C_{dl}$$

where C_s is the specific capacitance. In this work, 0.04 mF cm^{-2} was adopted as the value of C_s based on previously reported OER catalysts in alkaline solution.[1]

For comparison, ~5 mg of RuO_2 was ultrasonically dispersed in a mixture of 500 µL ethanol, 450 µL water and 50 µL of 5% Nafion and the dispersion was transferred onto on the carbon cloth substrate electrode (denoted as RuO_2) for electrochemical measurements.

Arrhenius Apparent Activation Energy Determination.

The electrochemical activation energy (E_a) for OER can be estimated by the Arrhenius relationship:[2]

$$\log j = \frac{-Ea}{In10 \times R \times T} + \text{const}$$

where j is the current density at η = 300 mV, R is the universal gas constant (8.314 j K^{-1} mol^{-1}), T is the temperature. The E_a can be extracted from the slope of Arrhenius plot. We considered the temperature effect when converting applied potential to relative hydrogen electrode potential.

Supporting Data

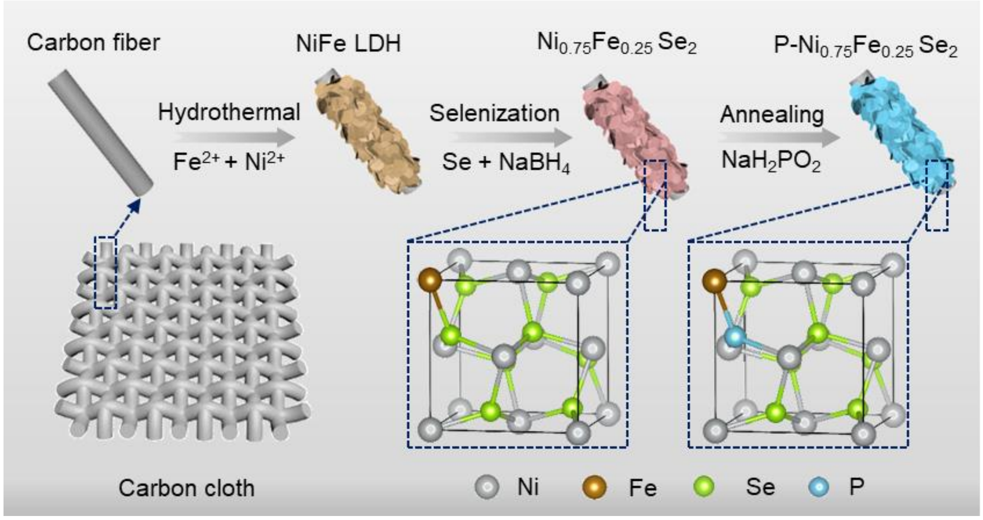

Scheme S1. Scheme for the fabrication processes of P-$Ni_{0.75}Fe_{0.25}Se_2$ supported on carbon cloth.

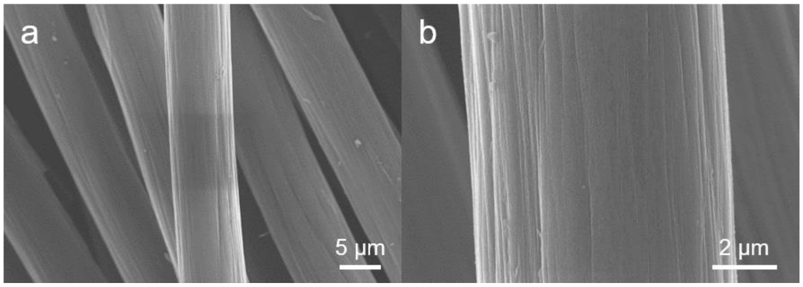

Figure S2. SEM images of pristine carbon cloth.

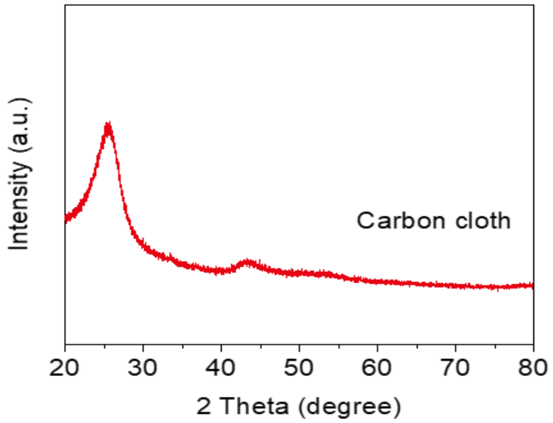

Figure S3. XRD pattern of pristine carbon cloth.

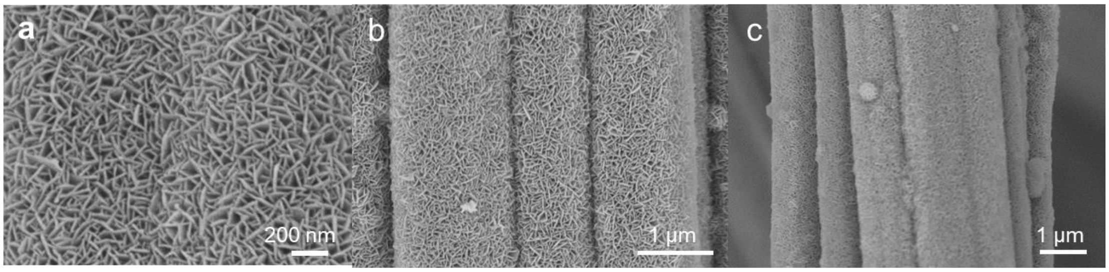

Figure S4. SEM images of NiFe LDH.

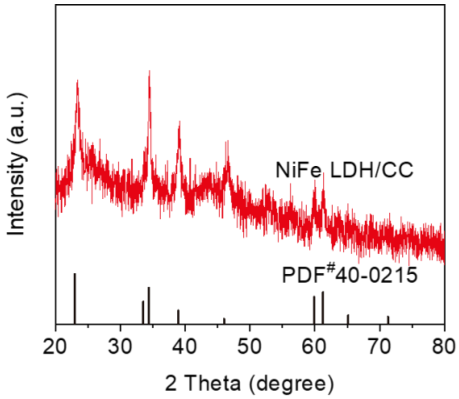

Figure S5. XRD pattern of pristine NiFe LDH.

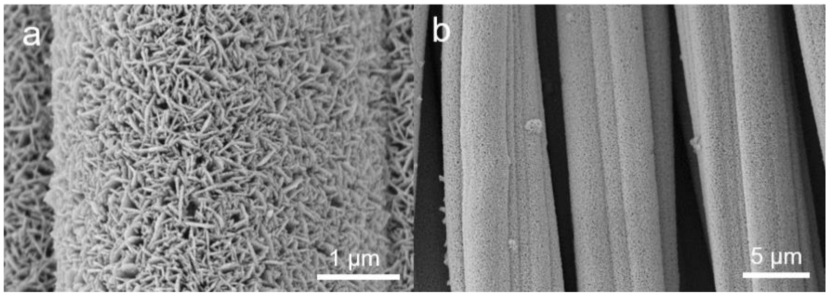

Figure S6. SEM images of $Ni_{0.75}Fe_{0.25}Se_2$.

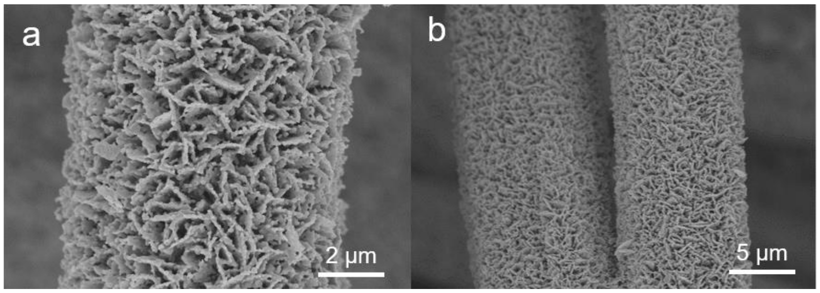

Figure S7. SEM images of P-$Ni_{0.75}Fe_{0.25}Se_2$.

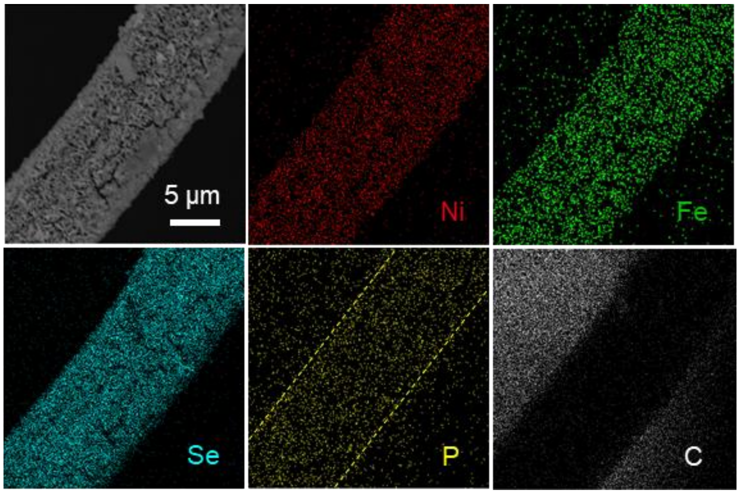

Figure S8. A SEM image and corresponding elemental mapping images of P-$Ni_{0.75}Fe_{0.25}Se_2$.

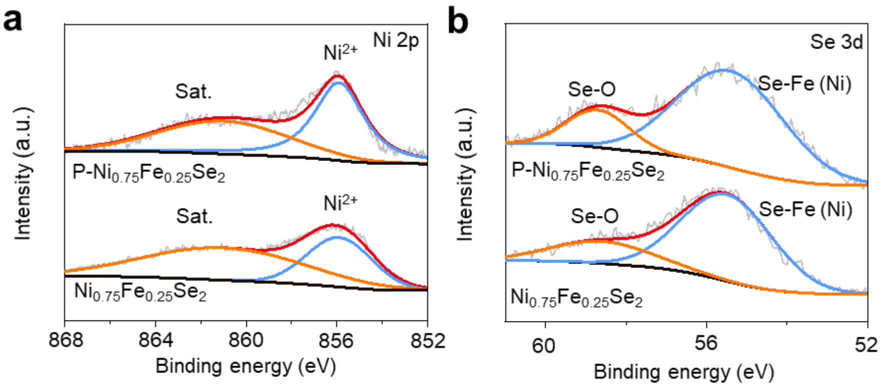

Figure S9. (a-b) High-resolution XPS spectra of $Ni_{0.75}Fe_{0.25}Se_2$ and P-$Ni_{0.75}Fe_{0.25}Se_2$: a) Ni 2p, b) Se 3d.

For the spectra of Se 3d, the peak at 55.5 eV can be assigned to Se-Fe (Ni) while the other peak at 58.6 eV belongs to Se-O due to the surface oxidation.

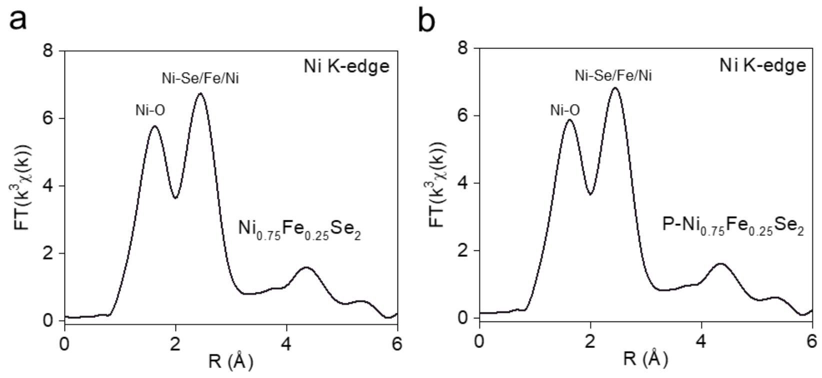

Figure S10. Fourier transforms (FT) EXAFS Ni K-edges spectra of $Ni_{0.75}Fe_{0.25}Se_2$ (a) and (b) P-$Ni_{0.75}Fe_{0.25}Se_2$.

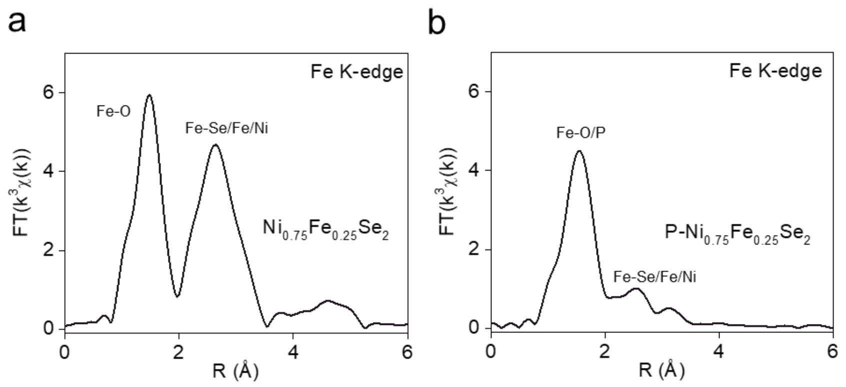

Figure S11. Fourier transforms (FT) EXAFS Fe K-edges spectra of $Ni_{0.75}Fe_{0.25}Se_2$ (a) and (b) P-$Ni_{0.75}Fe_{0.25}Se_2$.

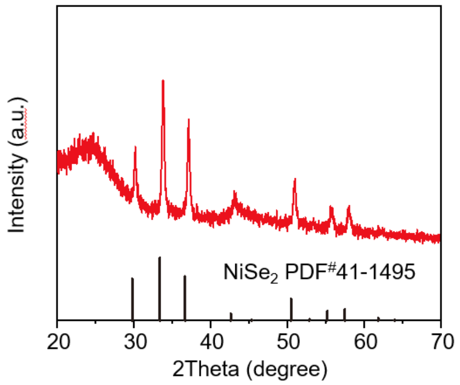

Figure S12. XRD pattern of P-$Ni_{0.75}Fe_{0.25}Se_2$ after OER stability test.

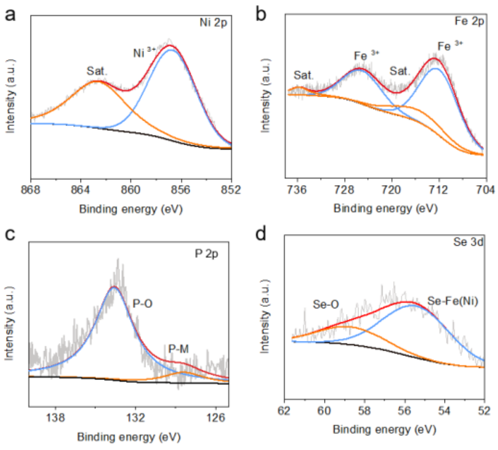

Figure S13. High-resolution XPS spectra of a) Ni 2p, b) Fe 2p, c) P 2p, and d) Se 3d of P-$Ni_{0.75}Fe_{0.25}Se_2$ after stability test.

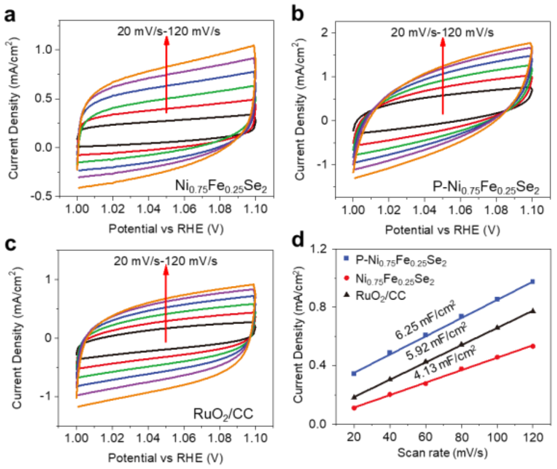

Figure S14. (a-c) Cyclic voltammograms at various scan rates of 20, 40, 60, 80, 100 and 120 mV s^{-1} for a) $Ni_{0.75}Fe_{0.25}Se_2$, b) P-$Ni_{0.75}Fe_{0.25}Se_2$. c) RuO_2/CC. (d) Corresponding double-layer capacitance.

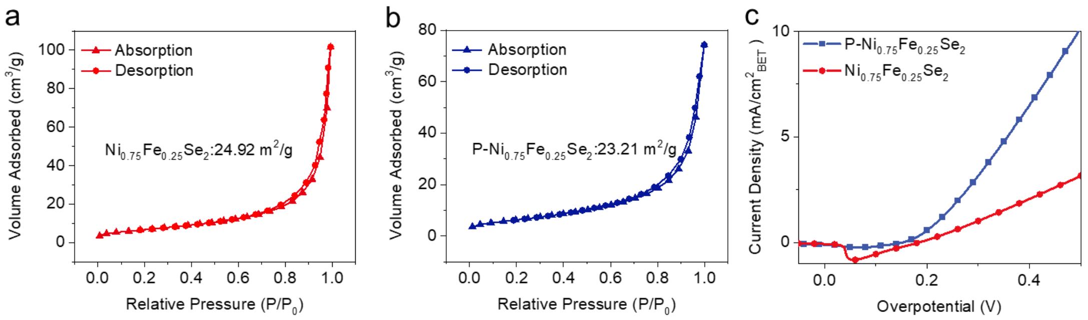

Figure S15. N_2 sorption isotherms of (a) $Ni_{0.75}Fe_{0.25}Se_2$ and (b) P-$Ni_{0.75}Fe_{0.25}Se_2$. (c) The polarization curves normalized by the BET surface area.

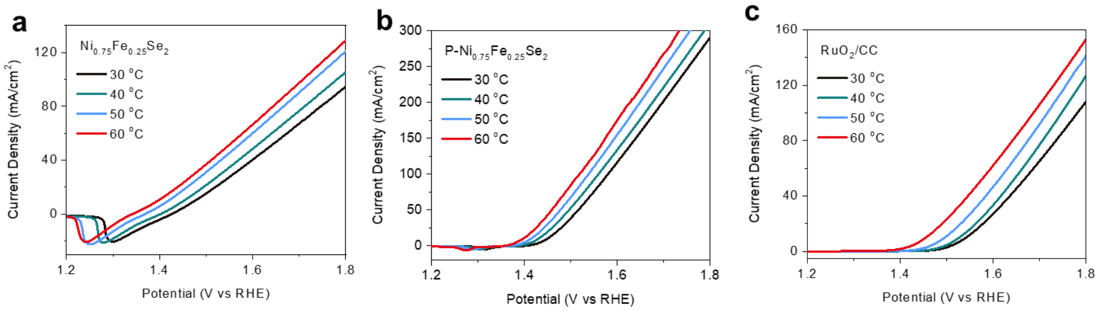

Figure S16. OER polarization curves tested at different temperature. (a) $Ni_{0.75}Fe_{0.25}Se_2$, (b) P-$Ni_{0.75}Fe_{0.25}Se_2$ and (c) RuO_2/CC in 1M KOH with scan rate of 5 mV s^{-1} at 30 °C, 40 °C, 50 °C, and 60 °C, respectively.

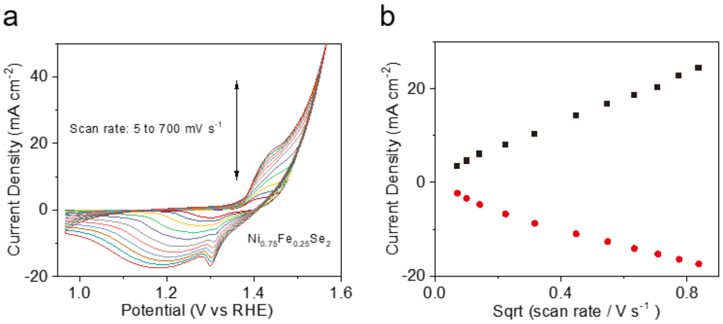

Figure S17. Analysis of $Ni_{0.75}Fe_{0.25}Se_2$ in Laviron equation. (a) CVs of $Ni_{0.75}Fe_{0.25}Se_2$ with scan rates from 5, 10, 20, 50, 100, 200, 300, 400, 500, 600 to 700 mV s^{-1}, in 1M KOH. (b) The plot of the redox peak currents densities versus the square root of scan rates.

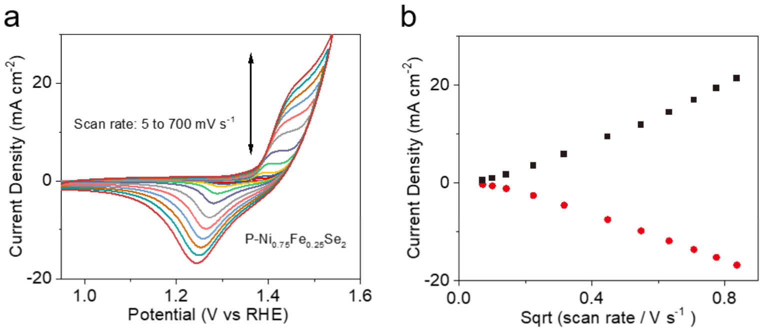

Figure S18. Analysis of P-$Ni_{0.75}Fe_{0.25}Se_2$ in Laviron equation. (a) CVs of $Ni_{0.75}Fe_{0.25}Se_2$ with scan rates from 5, 10, 20, 50, 100, 200, 300, 400, 500, 600 to 700 mV s^{-1}, in 1M KOH. (b) The plot of the redox peak currents densities versus the square root of scan rates.

Table S1. The ICP analysis data of P-$Ni_{0.75}Fe_{0.25}Se_2$.

Element	Concentration (mg/L)	Ratio
Fe	15.98	1
Ni	51.24	3
Se	183.57	8
P	0.28	0.03

Table S2. The composition of P-$Ni_{0.75}Fe_{0.25}Se_2$ determined by XPS.

Element	Fe	Ni	Se	P	O	C
Content (%)	2.61	4.90	17.61	1.35	30.81	42.72

Table S3. Comparison of the OER electrocatalytic performance in 1 M KOH of nickel-iron based electrocatalysts reported.

**Electrocatalysts**	**Overpotential at 10 mA cm^{-2} (mV)**	**Tafel slope (mV dec^{-1})**	**Ref.**
P-$Ni_{0.75}Fe_{0.25}Se_2$	185	27.2	This work
NiSe	270[a]	64.0	[3]
$NiSe_2$	250	38.0	[4]

Ni_3Se_2	270	142.8	[5]
NiFeP	219	32.0	[6]
NiFe LDH	181	42.3	[7]
S-$NiFe_2O_4$	267	36.7	[8]
(NiFe)PS_3	223	41.7	[9]
$Ni_{0.65}Fe_{0.35}P$	270	60.0	[10]
$Ni_{0.75}Fe_{0.25}Se_2$	267	67.0	[11]
$Ni_xFe_{1-x}Se_2$-DO	195	28.0	[12]
NiSe-$Ni_{0.85}Se$	300	98.0	[13]
Ni_2P@$FePO_x$	205	67.0	[14]
NiSe@NiOOH	332^b	162.0	[15]
Fe-NiSe/FeNi foam	245^b	65.0	[16]

Note: ^{a} 20 mA cm^{-2}; ^{b} 50 mA cm^{-2}.

Table S4. The loading amount, mass activity and TOF of different catalysts.

Samples	Loading of catalyst (mg/cm^2)				Mass activity (A/g)	TOF (s^{-1})
	1	2	3	Average		
RuO_2		0.25		0.25	256.68	0.09
$Ni_{0.75}Fe_{0.25}Se_2$	0.76	0.83	0.70	0.76	102.90	0.06
P-$Ni_{0.75}Fe_{0.25}Se_2$	0.67	0.76	0.73	0.72	328.19	0.18

Table S5. Fitting results of EIS for RuO_2, $Ni_{0.75}Fe_{0.25}Se_2$ and P-$Ni_{0.75}Fe_{0.25}Se_2$.

Samples	R_s (Ω)	R_{ct} (Ω)
RuO_2	2.75	165.00
$Ni_{0.75}Fe_{0.25}Se_2$	3.75	42.60
P-$Ni_{0.75}Fe_{0.25}Se_2$	3.70	8.55

References

[1] C. C. L. McCrory, S. Jung, J. C. Peters, T. F. Jaramillo, *J. Am. Chem. Soc.* **2013**, *135*, 16977.

[2] Marcel. Risch, K. A. Stoerzinger, T. Z. Regier, D. Peak, S. Y. Sayed. Y. Shao-Horn, *J. Phys. Chem. C* **2015**, *119*, 18903.

[3] C. Tang, N. Cheng, Z. Pu, W. Xing, X. Sun, *Angew. Chem., Int. Ed.* **2015**, *127*, 9483.

[4] I. H. Kwak, H. S. Im, D. M. Jang, Y. W. Kim, K. Park, Y. R. Lim, E. H. Cha, J. Park, *ACS Appl. Mater. Interfaces* **2016**, *8*, 5327.

[5] A. Swesi, T. J. Masud, M. Nath, *Energy Environ. Sci*. **2016**, *9*, 1771.

[6] F. Hu, S. Zhu, S. Chen, Y. Li, L. Ma, T. Wu, Y. Zhang, C. Wang, C. Liu, X. Yang, L. Song, X. Yang, Y. Xiong, *Adv. Mater*. **2017**, *29*, 1606570.

[7] H. Yang, C. Wang, Y. Zhang, Q. Wang, *Sci. China Mater.* **2019**, *62*, 681.

[8] J. Liu, D. Zhu, T. Ling, A.Vasileff, S. Qiao, *Nano Energy* **2017**, 40, 264.

[9] Z. Liu, Y. Wang, R. Chen, C. Chen, H. Yang, J. Ma, Y. Li, S. Wang, *J. Power Sources* **2018**, 403, 90.

[10] Z. Liu, G. Zhang, K. Zhang, H. Liu, J. Qu, *ACS Sustainable Chem. Eng.* **2018**, *6*, 7206.

[11] L. Lv, Z. Li, K. Xue, Y. Ruan, X. Ao, H. Wan, X. Miao, B. Zhang, J. Jiang, C. Wang, K. K. Ostrikov, *Nano Energy* **2018**, *47*, 275.

[12] X. Xu, F. Song, X. Hu, *Nat. Commun.* **2016**, *7*, 12324.

[13] Y. Chen, Z. Ren, H. Fu, X. Zhang, G. Tian, H. Fu, *Small* **2018**, *14*, 1800763.

[14] F. S. Zhang, J. W. Wang, J. Luo, R. R. Liu, Z. M. Zhang, C. T. He, T. B. Lu, *Chem. Sci.* **2018**, *9*, 1375.

[15] X. Li, G. Han, Y. Liu, B. Dong, W. Hu, X. Shang, Y. Chai, C. Liu, *ACS Appl. Mater. Interfaces* **2016**, *8*, 20057.

[16] C. Tang, A. M. Asiri, X. Sun, *Chem. Commun.* **2016**, *52*, 4529.
